# Getting Old Well in Sub Saharan Africa: Exploring the Social and Structural Drivers of Subjective Wellbeing among Elderly Men and Women in Uganda

**DOI:** 10.3390/ijerph17072347

**Published:** 2020-03-31

**Authors:** Andrea Rishworth, Susan J. Elliott, Joseph Kangmennaang

**Affiliations:** 1 Department of Geography, Pennsylvania State University, State College, PA 16802, USA; 2 Department of Geography and Environmental Management, University of Waterloo, Waterloo, ON N2L 3G1, Canada; elliotts@uwaterloo.ca; 3 Department of Geography and Earth Sciences, UNC Charlotte, Charlotte, NC 27514, USA; jkangmen@uncc.edu

**Keywords:** subjective wellbeing, aging, Uganda, social determinants of subjective wellbeing, environment

## Abstract

While literature attempts to explain why self-reported subjective wellbeing (SWB) generally increases with age in most high-income countries based on a social determinants of a health framework, little work attempts to explain the low levels of self-report SWB among older persons in sub-Saharan Africa. Using the 2013 Uganda Study on Global Aging and Health with 470 individuals, this research examines (i) direct and indirect effects of age on SWB through social and structural determinants, and (ii) how direct and indirect effects vary by gender. Results show a significant direct and negative effect of age on SWB (*β* = 0.42, *p* = 0.01). Six indirect paths were statistically significant and their indirect effects on wellbeing varied by gender. Providing support, education, working status, asset level, financial status and financial improvement were significantly positively associated with men’s SWB, whereas younger age, providing community support, participating in group activities, number of close friends/relatives, government assistance and all socio-economic variables were significantly positively associated with women’s SWB. Strategies to address gendered economic, social and political inequalities among and between elderly populations are urgently needed.

## 1. Background 

As older populations increasingly comprise a larger share of the global population, countries and international organizations are seeking ways to enhance the subjective wellbeing (SWB) of older populations [1,2]. This growing attention is due in part to the recognition that SWB is associated with positive health outcomes, such as improved immune responses [3] and the adoption of healthy behaviors [4], as well as increased longevity [5]. Numerous studies examining the association between age and SWB in high-income contexts consistently reveal that across individuals, countries and cultures in high-income western societies, SWB generally increases with age [6,7,8]. This relationship is typically depicted as a U-shape relation, whereby the highest levels of SWB are achieved during youth and old age [6,7,8]. These studies suggest that despite the challenges associated with aging—loss of social status, reduced income, the death of loved one—older people maintain and even increase self-report SWB [6,8,9]. 

However, recent literature from an expanded range of geographic contexts find the inverse relationship, whereby markers of illbeing increase with age in several low–middle income countries [8,10]. For instance, using Gallup World Poll data from more than 160 countries, Steptoe, Deaton and Stone found progressive reductions in wellbeing with age in former Soviet Union, Eastern Europe, Latin America and sub-Saharan Africa (SSA) [8], suggesting important geographic differences in the way SWB unfolds in old age. Graham and Pozuelo extend these insights by examining why some nations, and people within them, have SWB turning points that are much earlier while others turn much later [10]. In so doing, Graham and Pozuelo find that despite the consistency in the U-shape pattern across people, countries and well-being dimensions, on average, the curve turns earlier for happier people and for people in happier places [10]. Moreover, the study found that those same respondents experience a drop in stress levels earlier in life [10]. An important point from this study is that individuals who are naturally cheerful or happy and live in places that have environments conducive to higher levels of well-being, due to social safety nets, health care systems, and supportive government systems, not only tend to have more years of wellbeing, but also better years of wellbeing. In contrast, individuals in very difficult places, marked by poverty, socioeconomic instability and political unrest, among other factors, tend to have very limited upward turns in wellbeing [10]. 

These findings are particularly important in the sub-Saharan Africa context, since seniors in the region not only have the lowest reported levels of SWB in old age, but also the greatest prevalence of worry, stress and unhappiness with increasing age [8,11]. This trend is concerning given that SSA is expected to see the largest absolute rise of its elderly population, increasing 15-fold from 46 million to 694 million by 2100 [12]. While current literature from high-income Western societies highlight a range of environmental factors, such as social support, economic assets and political resources, at the individual, community and structural level are important for the maintenance and promotion of subjective wellbeing in old age [13,14,15], little attention has sought to examine how these factors contribute to old age SWB declines in SSA.

Examining these issues in the Ugandan context is particularly important given that the current cohort of older adults are living longer, yet with minimal social, economic or institutional assistance [16,17]. In Uganda, seniors are affected by a range of interpersonal, community and structural factors, such as waning social support and interpersonal connections, care for patients with human immunodeficiency virus/acquired immunodeficiency syndrome (HIV/AIDS), poor health infrastructure and growing economic disparities [18,19,20]. At the same time, studies indicate that seniors are particularly challenged due to declines in socioeconomic status, household wealth and pension support [21,22]. These issues are acutely concerning for older women given the cumulative disadvantages in access and use of key social and economic resources they experience over their lives [21,22]. Despite the importance of these environmental factors for SWB in older ages, little knowledge exists on the determinants of SWB in Uganda. Since the promotion of SWB in old age is essential for one to age well, this research draws on the World Health Organization (WHO) Commission on Social Determinants of Health Framework to examine the determinants of seniors SWB in Uganda. This research specifically uses secondary data from Uganda to examine (i) direct and indirect effects of age on SWB through a range of moderating social and structural determinants, and (ii) how these direct and indirect effects vary by gender.

## 2. Aging and Subjective Wellbeing 

Subjective wellbeing is defined as “a person’s cognitive and affective evaluations of his or her life” [15]. In the past decade, an increasing consensus has emerged surrounding the need to measure distinct aspects of SWB, namely, evaluative, hedonic and eudemonic components [15]. Evaluative wellbeing captures the cognitive assessment of one’s life satisfaction in global (e.g., life as a whole) and domain specific areas of life (e.g., work, relationships, etc.) [15]. Hedonic wellbeing addresses the affective everyday feelings or moods associated with one’s life [23], and is considered positive when emotions, moods and feelings are pleasant (e.g.., joy, elation, affection, etc.), or negative when emotions, moods and feelings are unpleasant (e.g., guilt, anger, shame, etc.) [15]. Lastly, eudemonic metrics refer to judgements about the meaning and purpose of one’s life [24]. Due to the diversity of the construct, several questions are commonly employed to query aspects of eudemonic wellbeing [24]. While these three types of wellbeing are distinct due to the level of cognitive processing required to make these different evaluations, together they provide a unified way to evaluate the SWB of an individual or population. 

Current studies from high-income countries indicate that a range of factors influence subjective evaluations of wellbeing in old age [14,15]. Social groups, relationships and support systems are thought to be strongly associated with SWB in later life [25,26,27]. While seniors experience physical functional declines and a reduction in activities with increasing age, social support systems (e.g., family, friends, clubs, groups) can enhance social relationships, promote happiness and generate SWB in old age [25,26]. In one way, supportive relationships can buffer negative psychological outcomes [28], reduce incidence of depression and mental discomfort [29] in old age, while in another way, social relations can provide a resource to help fulfill emotional goals and maintain functionality in later life [29]. An important point from these studies is that despite the physiological challenges associated with aging, social relationships can help alleviate some of the psychosocial challenges in old age and thus help to promote SWB [28,29]. 

Marital status and marital transitions are important for SWB in old age [14,30,31]. Studies consistently reveal that being married is strongly and significantly related to higher SWB since marriage provides social supports and the accumulation of resources that can directly and indirectly affect SWB in later life [15,30,31]. A meta-analysis of 286 studies found that due to the material and affective factors associated with marriage, older married adults have a higher SWB rating compared to their unmarried peers [7], while transitioning out of a marriage in later life, due to divorce or death, is associated with decreases in SWB [32,33]. While marriage impacts the SWB of both men and women, studies indicate that the deterioration of a marriage bond is particularly detrimental for the SWB of men [34,35]. Yet, in Uganda, where older men are more likely to remarry after the death of a partner compared to older women, due in part to patriarchal inheritance practices, it is unclear whether marriage will have the same effect on SWB outcomes [36]. 

The importance of socioeconomic status, such as income and education, for outcomes for SWB in old age is mixed [37]. On one hand, most studies suggest that seniors in high-income brackets have better outcomes of SWB compared to their financially constrained counterparts, due to their ability to directly access financial resources and indirectly draw down on a range of cultural and social resources [38,39]. On the other hand, while education significantly impacts SWB during youth and adulthood, studies consistently demonstrate that education has a small to negligible influence on SWB in old age [7,40]. Yet, in Uganda, where only some seniors, most often males, are educated, the effects of education on SWB are unclear. 

The role of gender on SWB in old age is unique and is found to vary across the life course. While women in their adolescence and adulthood, on average, report higher levels of SWB compared to men [41], in old age, older women often report lower levels of SWB than older men due to disadvantages in income, social relations and socioeconomic status that accrue in later life [7,42]. Yet, other studies suggest that older men report lower levels of SWB since they have fewer tasks from which to gain happiness and are more prone to difficulties developing and continuing intimate relationships that often protect against low SWB [38,43]. In many SSA contexts, where women consistently have less access and control over resources across the life course, the role of gender on older women’s SWB outcomes is likely exacerbated. 

Geographic variations in location of residence also influences SWB outcomes in old age. Studies commonly indicate that compared to seniors in urban localities, those in rural areas have significantly enhanced SWB compared to their urban counterparts due to the positive effects of social networks and support systems [44,45,46]. However, as younger populations increasingly migrate to urban localities in Uganda [18,19,20], it is unclear whether this relationship between SWB and urban-rural localities will remain consistent. 

This growing body of literature provides important insight into the individual, community and institutional socioeconomic and demographic factors that both positively and negatively impact SWB in old age. That said, despite the importance of SWB in old age, its analysis remains limited in low-middle income countries (LMICs) [47]. The few studies exploring SWB among elderly in SSA (i.e., South Africa, Nigeria, Ghana), reveal that seniors with low socioeconomic status, few social supports and/or identify as a woman, are more likely to report poor SWB compared to their economically advantaged, socially connected, male counterparts [48,49,50,51]. An important point from these investigations is that more work needs to tease out the role of particular determinants in the relationship between SWB and aging across a wider range of geographic contexts [50,51]. These statements are buttressed by recent academic and systematic reviews calling for more work to investigate the possible moderating factors and pathways that shape SWB in old age, particularly in under-resourced contexts where substantive gaps in knowledge exist [13,52]. 

This paper begins to address this gap in the context of Uganda, where the older population is projected to increase almost five-fold from 1.3 million to 5.5 million between 2010 and 2050 [12,53], yet with little to no institutional support to enable the older population to ‘age well’ with enhanced subjective wellbeing. Since the links between SWB and its determinants are context-dependent, shaped by the particularities of time and space [54], this study draws on the World Health Organization Commission on Social Determinants of Health to address two interrelated objectives. First, this paper aims to examine the direct and indirect effects of age on SWB through each moderating factor, and second, to compare how these direct and indirect effects vary by gender. By exploring these relationships, this research provides insight into the multidimensional drivers of SWB in Uganda, contributes new knowledge on the gendered differences in SWB and illuminates the adaptability of the WHO Commission on Social Determinants of Health Framework to the study of SWB in old age. 

## 3. Framing Aging and SWB in SSA

**Figure 1:**
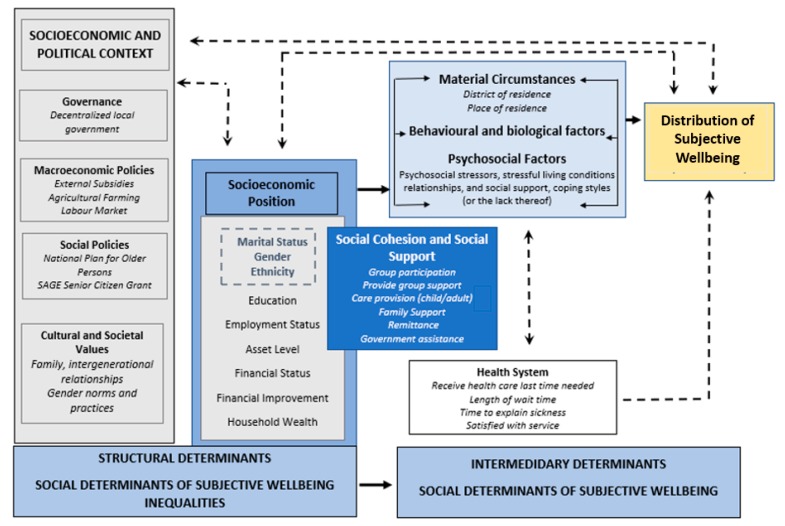
The Social Determinants of Elderly Subjective Wellbeing in the Central Region of Uganda. Adapted from the World Health Organization (WHO) Commission on Social Determinants of Health Framework [56].

This research adopts and extends the framework developed by the World Health Organization Commission on Social Determinants of Health (CSDH) (Figure 1). This framework recognizes the role that social factors play in shaping the health and wellbeing of individuals and populations. By conceptualizing outcomes of poor health and wellbeing as products of economic and social inequality, the CSDH provides the conceptual space to begin examining how the relationship between age and SWB is moderated by various socio-economic and demographic factors. 

According to the CSDH, understanding inequalities in health and wellbeing requires examining the origins of social inequality and the pathways through which these inequalities emerge [55,56]. Indeed, older populations in SSA have the lowest global life expectancy (e.g., 38-year difference compared to high-income countries) and highest disease burdens (both chronic and infectious) in old age, due to the cumulative impacts of inequalities across the life course [1,55]. Yet even within this demographic, inequalities exist [1]. Older populations in socially and economically deprived positions are exposed to more health risks, experience greater health problems and have shorter life expectancies—all of which negatively impact happiness, life satisfaction and subjective wellbeing [1,57]. 

Since social and economic inequalities stem from the discriminative impacts of structural and individual allocation and control of resources to individuals and populations [56], examining how these inequalities impact SWB outcomes among elderly populations in SSA is imperative in order to address their needs [1]. For instance, when the allocation of resources for populations at the household, community and country level is exercised in a manner that neglects elderly populations, it creates disparities in access to economic and social resources and creates long-term inequalities. At the same time, however, individual attributes and life choices simultaneously contribute to disparities in one’s socio-economic position. Together, these factors work to create inequalities to health and wellbeing [58,59]. 

Disparities in the health and wellbeing of seniors are informed by two sets of determinants—structural and intermediary (Figure 1). Structural determinants comprise all social and political mechanisms (governance, macro-economic policy, social policy, public policy, social and cultural values) that generate, configure and maintain an individual’s socioeconomic position (i.e., income, education, occupation, gender) within hierarchies of power, prestige and access to resources [56]. In old age, these hierarchies increase in relevance due to the cumulative impact of inequalities experienced over the life course [1,56]. Overall, these structural determinants are the social processes that shape the underlying downstream intermediary social determinants of health (Figure 1) [59].

Intermediary social determinants of health comprise material conditions (e.g., living and working conditions, neighborhood, consumption abilities), psychosocial factors (e.g., psychosocial stress factors, social support or lack thereof), behavioral and biological factors (e.g., the distribution of nutrition, tobacco, alcohol, physical activity, genetic factors) and the health system itself. Together, these factors shape one’s exposure to health, enabling or compromising conditions that, over the life course, contribute to inequalities in health and wellbeing in old age [55,56]. 

Psychosocial factors, such as social support networks and social cohesion, interact with structural factors, either enhancing or restricting health and wellbeing outcomes. For instance, networks of relationships among groups of people, a high degree of reciprocal trust, and strong social norms can provide material and non-material benefits for mutual and collective gains [60,61], as well as buffer against the deleterious impacts to health and wellbeing among individuals in low-socioeconomic contexts since they serve as critical resources for the realization of needs among group members [60,62]. Interactions between structural and intermediary determinants then result in differentiations (inequalities) in health and wellbeing, which can feed back into the structural determinants of health and have a positive, negative or neutral influence on future generations [56]. 

This framework provides the conceptual space to study the social production of subjective wellbeing. Employing the CSDH not only addresses calls from the WHO for more engagement on the social determinants of wellbeing in old age [1,55], but also provides empirical evidence to begin addressing knowledge gaps identified by both the WHO Report on Aging and Health and the Global Strategy and Action Plan for Aging and Health [1,61]. Furthermore, the application of the CSDH framework to this case study lays a foundation on which to build future investigations. 

## 4. Aging in Uganda 

While Uganda’s population is aging, 98% of elderly have no access to pensions or financial security [17]. Due in part to the lack of financial support, 85% remain engaged in informal subsistence farming, and/or depend on communal and intergenerational relations for both financial and social support [63]. Although Uganda is piloting the Senior Citizens Grants under the Irish Aid Social Assistance Grants for Empowerment (SAGE) Scheme, income support grants have yet to be equitably rolled out across the country [64]. Uganda has the highest youth unemployment rate in SSA, which is driving the high rates of rural-urban migration of younger populations and consequently fracturing intergenerational social systems [17]. Compounding this situation is the high HIV prevalence (7.1%), which has left many elders to care for orphaned and/or sick and vulnerable kin or sick adult children and family members [16,63]. Even though Uganda has a National Plan for Older Persons—one of the few SSA countries to do so—that aims to create an environment where older persons can live in a secure and dignified environment that fulfills their needs through various priority areas (e.g., economic empowerment, social security, food and nutrition, health care, education, psychosocial support, water, sanitation and shelter), the decentralization of government services has resulted in service fragmentation and little to no implementation of support for this population [16].

## 5. Research Design and Methods

Data from the 2013 Uganda WHO Multi-Country Study on Global Aging and Adult Health (SAGE), second wave (2013), were used for this research. SAGE compiles comprehensive longitudinal data on the health and wellbeing of adult populations and documents the aging process across different countries (i.e., China, Ghana, India, Mexico, Russia, South Africa, Uganda). The use of data from the 2013 Uganda SAGE survey was deemed appropriate as it enables comparability across participating SAGE countries and generates opportunities for similar investigation over time. The Ugandan SAGE Wave 2 Study on Global Aging and Health (SAGE) Wellbeing of Older People Study used a multistage stratified cluster sampling design (for further details on the survey methodology see [65,66]. The sample aged 50 and above [as recommended by the WHO, the international network of field sites with continuous Demographic Evaluation of Populations and their Health Network (INDEPTH network) for African contexts and other SAGE studies] [1,21,67,68] was selected from existing databases. 470 people were interviewed: 302 in the rural Masaka District and 168 in the urban Wakiso District. Of the 470 people interviewed, 176 were men and 294 were women. The survey was conducted from February to September 2013 and was implemented by a physician, nurse or non-clinically trained individual in homes or a central location in a village. All survey questions were read to the participants in Luganda (for further information on survey implementation see [69]. The survey was implemented sequentially from the rural to the urban site. While this dataset is challenged due to its lack of breadth, it does however provide a depth of insight into the multifarious factors impacting aging populations in Uganda’s Central Region. 

Ethical clearance for the study was given by the Uganda Virus Research Institute Science and Ethics Committee and the Uganda National Council for Science and Technology. This study uses publicly available data (www.who.int/healthinfo/systems/sage) and is not involved in any contact with the participants, therefore further ethical approval was not considered necessary. We abided by the data access agreement that ensures that data is only used in an ethical manner. 

The data collection tools were adapted from the WHO Multi-Country Study on Global AGEing and Adult Health (SAGE) and have been used in multiple settings [65,66]. The SAGE household and individual questionnaires cover a wide range of topics related to demographic and household characteristics, both objective and subjective measurements of health and wellbeing and a module focused on caregiving and care receiving. Independent variables cover a wide range and are conceptually relevant to the aging process [15,17,68]. Satisfaction with several life domains was used to capture the SWB of respondents.

The dependent variable used in the analysis was subjective wellbeing (SWB), calibrated through the World Health Organization 12-item quality of life brief (WHOQOL-Bref) questionnaire, shown to be a valid measure of SWB among the elderly [70,71,72]. The analysis specifically examined evaluative wellbeing. Questions addressed aspects related to quality of life, life satisfaction and affect in four domains: physical, psychological, social relationships and environment. Respondents were asked whether they had enough energy or money, and whether they were satisfied with their health, self-image, ability to perform daily activities, personal relationships, living conditions, etc. SWB scores ranged from 0–13.25, whereby higher values reflect a poorer subjective wellbeing score and lower values reflect a better subjective wellbeing score. Confirmatory factor analysis was performed on these items, using principal-factors extraction and orthogonal varimax rotation. Factors with eigen values greater than 1.0 [73,74] were retained. The eigenvalues in decreasing order were plotted to identify the scree, i.e., the portion of the graph where the slope of decreasing eigenvalues approaches zero [75]. Although we did have an explicit test of a single factor solution, the first item had an eigenvalue of 5.19, which explained 52% of the variation, large enough for us to be reasonably confident that all 10 items are trapped on a single dimension. We obtained high overall reliability (α = 0.91) and high overall sampling adequacy (kmo = 0.89). The main independent variable is age of respondent. 

Structural equation modeling (SEM) was used to examine how the link between age and SWB among older adults is moderated by structural and intermediary social factors. SEM was chosen as it provided the ability to analyze structural relationships between measured variables (i.e., observable, measured variables) and latent constructs (i.e., variables that are not directly observed but are rather inferred from other observable variables) as well as estimate the multiple interrelated dependent variables in a single analysis [76]. Guided by the CSDH, structural factors comprised all socioeconomic variables, the demographic variable ‘marital status’, and the care variable ‘receive government assistance’. Intermediary social factors comprised all of the health care variables, all of the care variables, except ‘receive government assistance’, and the demographic variables ‘residence/location’ and ‘religion’. These multiple potential factors were then tested to examine how they moderate the relationship between age and SWB. Using structural equation modeling provided the total effect, or c path, direct effect and indirect effects of age with wellbeing through each moderator. This method also allows an examination of the extent to which the moderators independently contribute to an explanation of the association of the focal variable with the outcome variable as well as a comparison between moderators. 

## 6. Results

Women in the sample were slightly younger than men and reported lower SWB scores (6 and 7, respectively; Table 1). 73% of women and 58% of men provided care for children, while only 13% of women and 10% of men provided care for adults. More women received family assistance (64% and 45%, respectively) and remittances from children (36% and 18%, respectively). However, more women reported waiting longer for health care (51%; 40%, respectively), having less time to explain sickness (17% and 10%, respectively) and being less satisfied with health care (12% and 7%, respectively). More women were poor compared to men (39% and 25%, respectively), whereas more men were rich compared to women (40% and 30%, respectively). Most men and women reported currently working (83% and 79%, respectively). Primary education was reported as the highest level among most women and men (57% for both). Yet, more men received a secondary education compared to women (27% and 13%, respectively), and more women had no education compared to men (23%; 10% respectively). More women were not married (12%; 88%, respectively), whereas more men were married (63%; 37%, respectively). Most men and women reported residing in rural locations (69%; 62% respectively). However, slightly more women reported living in an urban location compared to men (38%; 31% respectively). 

In the first stage of the multivariate analysis, we employed linear regression to examine the links between age and SWB controlling for only demographic factors (Table 2). The results show that age had a strong and negative association with SWB (β = 0.42, *p* = 0.01). Among the demographic factors, gender, marital status and asset levels were significantly associated with wellbeing, albeit with positive and negative effects. When the first group of factors (social relationships and care responsibility factors) were controlled for, the association between age and SWB remained significant (β = 0.38, *p* = 0.01) although the effect size reduced. Factors such as providing community support, group membership and the number of close friends/relatives were significantly and positively associated with both age and SWB. However, caregiving responsibilities, such as providing care for an adult or a child, were only negatively associated with SWB. The association between age and subjective wellbeing remained robust (β = 0.42, *p* = 0.01) after we controlled for healthcare, social-economic and financial variables in the final model.

**Figure 2:**
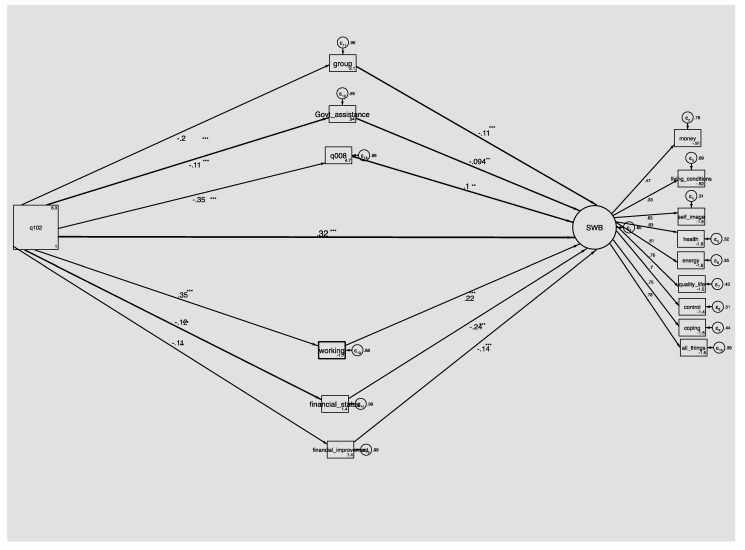
Indirect pathways between aging and subjective wellbeing in Central Uganda. SWB = subjective wellbeing in decreasing order, q101 = age, q008 = district of residence, q112 = religion, q101 = gender. ** *p* < 0.01, *** *p* < 0.001

Subsequent mediation testing [77] revealed that the direct effect (c) of age on wellbeing was significant (β = 0.33, *p* = 0.006). Six of the indirect paths were statistically significant (non-significant results are not displayed in the figure) and had positive effects but did not completely mediate the negative relationship between age and SWB (Figure 2). These include social relationship variables, such as belonging to a group (β = 0.02, *p* = 0.01), and receiving government support (β = 0.009, *p* = 0.01). Socioeconomic indirect paths include currently working (β = 0.07, *p* = 0.01), and better financial status (β = −0.03, *p* = 0.01), while none of the demographic factors were significant. This suggests that the effects of age on wellbeing operate through indirect effects on social support, socioeconomic factors as well as functional ability in terms of undertaking work. 

We further disaggregated the analysis by gender in order to examine the differential gendered effects of the aging process (columns 2 and 3 in Table 2). The significant factors positively associated with men’s SWB include providing support to others, education, working status, asset level, good financial status and financial improvement over the previous years. Among older women, the significant factors positively associated with SWB include younger age, providing community support, participating in group activities and higher number of close friends/relatives, government assistance and all of the socio-economic variables. Interesting, location of residence, was not a significant factor for either men or women, despite its ability to undermine access to key determinants of SWB, such as healthcare or employment opportunities. 

## 7. Discussion 

By examining the direct and indirect effects of age on SWB through moderating social and structural determinants, and exploring their gendered variation, this study provides important insight into the multidimensional social-structural determinants of SWB in old age in a SSA context. In terms of social determinants, the findings highlight that social relationships (e.g., community support, group participation) positively moderate the age–SWB relationship, consistent with findings from both high- and low-income countries, likely due to seniors’ need for social support and engagement in old age [26]. Yet, in Uganda, like in many other SSA countries, where formal social support is negligible, the results suggest that engaging in the provision of informal support through personal networks (see Table 1) may be even more important for physical and psychosocial health than in other high-income contexts [78]. Social supportive networks in many SSA countries not only act as social buffers and support for interpersonal connections in which to obtain joy, satisfaction and happiness in old age [79], they also mitigate the deleterious impacts of socioeconomic inequalities as they provide a means to obtain basic necessities including food, water and clothing [80].

Nevertheless, caregiving responsibilities significantly and negatively influenced SWB for women (−0.11) and men (−0.12) but had no effect on age. This suggests that engaging in caregiving responsibilities among men and women negatively impacts their SWB (see Table 1), possibly through economic, social and physical costs associated with additional responsibilities [81]. This finding supports other literature in SSA that demonstrates that caregiving may lead to increased physical and somatic complaints [82], and create psychosocial implications, especially where care is being provided with few financial resources [83]. Yet, these findings run contrary to other literature that suggests caregiving has positive outcomes for older people due to its capability to build obligatory exchanges, strengthen family ties and provide a means to access other types of care for themselves, like assistance with household chores or other daily tasks [84]. Given these diverse responses, more work is needed to tease apart the bi-directional relationships between SWB and caregiving/receiving among both older men and women in SSA. 

In terms of structural determinants, the results reveal that economically deprived elders have lower SWB evaluations compared to their economically advantaged counterparts, akin with other literature [7,13]. As one could expect, poorer evaluations of SWB are concentrated among individuals with lower socioeconomic status in terms of being unemployed, low wealth levels and worsening financial status (see Table 1). This finding underscores the likely cumulative impacts of economic inequalities experienced over the life course, and exposes inequalities in SWB, even in this relatively homogenous population where poverty is rife [52,53]. Moreover, the significant positive pathway between marital status and SWB, but not with aging, is revealing and highlights the importance of marriage for positive evaluations of SWB in later life, consistent with other studies [30,32,34]. Our findings, however, illuminate that irrespective of age, marriage in itself is positively and directly associated with SWB, perhaps by way of its ability to fulfil basic and universal human needs, provide companionship and freedom from loneliness, or through its ability to promote effective coping in the face of adversity [30]. 

Lastly, the findings uncover important gender variations in the social determinants of SWB that in part, are likely influenced by particular gender identities, roles and responsibilities that inform access to and control over resources over the life course [85]. In Uganda, patriarchal structures and deep-rooted attitudes regarding roles, responsibilities and identities of men and women mean women are responsible for maintaining and reproducing the household and community by managing a larger share of the domestic labor compared to men over their life course [36]. The results suggest that in old age, women derive more positive effects from participating in groups and providing community support, which may moderate some of the negative interactions between age and SWB. This finding aligns with prior work that demonstrates since women’s roles are tethered to the household, they are better able to form strong ties with other community members that afford them economic and social security, while men typically have weaker social ties as a result of working outside of the home and community [86]. Considering research on older adults’ social relationships in LMICs, particularly in SSA, is limited [87], further work needs to examine how different types of social relationships are associated with the wellbeing of older men and women in SSA [78].

Several other determinants were found to significantly and positively moderate the age–SWB relationship for women. These included social determinants such as their number of close contacts and various healthcare factors (i.e., healthcare receipt last time needed, had time to explain sickness at the health facility and satisfaction of health care services), and the structural determinant—receipt of government assistance. The importance of close contacts for women may represent how women’s longer lives on average, mean they are less likely to remarry in old age compared to men, and as a result, derive more happiness and support from the connections with close contacts than men [88,89]. This may be due to gender norms that in part, deem post-menopausal women less sexually appealing than young widows due to their presumed infertility and prohibit women to re-marry in old age as a result of heteronormative ideals linking marriage with procreation [90]. Moreover, the value of government assistance for women’s SWB may underscore how women’s limited access to formal employment during their lives and lack of associated pensionable income compared to men, mean the receipt of government assistance provides financial assurance, potentially alleviating (some) stressful experiences in later life [91]. The significant and positive impact of healthcare factors for older women’s SWB may underscore elderly women’s greater health challenges compared to men—similar to other studies conducted in Uganda [91], or may be emblematic of women’s, on average, greater health-seeking behavior compared to men [92]. 

On the other hand, older men’s age–SWB was more strongly and positively moderated by education and asset level (0.31; 0.33 respectively) compared to women (0.23; 0.26), suggesting that older men derive more positive effects from having higher education and financial asset levels. Since Ugandan men have greater access to formal education and formal income and have more control over household resources than women [16] (see Table 1), these gender advantages in education and employment likely help to explain why these factors play such an influential role in SWB evaluations. These findings are similar to those from Ethiopia that found that literacy rates were only associated with male but not female old age survival, suggesting gender differences in decision-making power place men in stronger positions to enjoy the benefits of literacy [93]. Our results may tell a similar story such that the gendered divisions of roles and responsibilities in Uganda which position women as caregivers and men as household providers [16], contribute to cumulative (dis)advantages in education and wealth over the life course [55] that become reflected in women’s lower and men’s higher SWB evaluations. This is consistent with the literature that highlights SSA as the only global region where women (ages 25 years+) have lower SWB evaluations than men [41,51], likely due to the accumulated inequalities in responsibilities and resources experienced in multiple spheres of life—education, employment, household reproduction—that combine to undermine SWB in old age. Considering that the results highlight that being a woman is directly and negatively associated with SWB, more work needs to examine how gendered advantages and disadvantages across the life course accumulate in differential outcomes of gendered wellbeing.

In addition to the aforementioned factors, marital status for men was more than twice as strong and positively associated with SWB (0.25) compared to women (0.12). This may suggest that since men in East Africa are found to have harder times coping with the aging process due to the loss of identity and employment status upon retirement, female spouses may cushion the aging process for men as they provide both psychological and livelihood support and facilitate opportunities for social connection [86]. This may also help explain why receiving family assistance for men was significantly and positively associated with enhanced SWB (−0.14), but not for women (0.02)—perhaps especially true in contexts where receiving family assistance provides some financial stability and psychological comfort where men get squeezed out of formal work or experience declines in ability to engage in informal economies [86]. Considering the paucity of research examining how marital status moderates the old age–SWB relationship in SSA or how this relationship varies by gender, this work extends current knowledge and underscores the need for more work at these intersections. 

Despite the relevance of our findings, there are a number of limitations worth noting. First, the data is cross-sectional. Thus, we are unable to make any causal inferences between any of our independent variables and SWB. Second, due to the design and implementation of the primary research, the study lacked a comparison group in which to make assessments. Yet, given the observational nature of the study, whereby participants were followed in their naturalistic setting, a comparison group was not necessary. Second, respondents were selected from existing cohort studies in the area, which could potentially introduce a selection bias. However, given the wide coverage of the study from which the samples are drawn, it is possible to have provided a fairly representative picture of older people living in the area. Third, self-report health and SWB measures are limited and may introduce bias. However, the use of the WHOQOL-Bref gives confidence that these results provide good insights into factors affecting older people’s SWB. Moreover, the measures used in the study have been well tested in a wide range of sociocultural and economic contexts [66] and are considered robust measures of SWB. Lastly, while the WHOQoL-Old would have been a better instrument to use, the WHOQoL-Bref was used because it was part of a larger survey comprising people under 50 years. 

## 8. Conclusions

In the context of population aging and declining rates of subjective wellbeing in LMICs, these results demonstrate the value of applying the CSDH framework to examine how socioeconomic and political contexts configure socioeconomic hierarchies that produce inequalities in old age SWB [52,56,59,60]. Considering the World Health Organization put forth its Global Strategy and Action Plan on Aging and Health (GSAP) to promote wellbeing in old age, yet to date, remains abstract and in need of operationalization and translation [94], the CSDH provides a way to begin explicating how different environmental conditions shape opportunities for wellbeing [59]. Since inequalities in SWB are identified as a leading global public health concern [1,59], these results suggest that more work needs to (i) examine the determinants of subjective wellbeing among older global populations, especially in LMIC contexts, (ii) investigate how determinants of SWB vary over time and space [95] and (iii) examine how inequalities shape life chances within and between elderly populations—all of which have important implications for policy and practice [13]. 

In the context of Uganda, the results suggest that policies and programs are needed that prioritize the provision of stable basic incomes and justified employment opportunities to improve livelihoods and reduce economic anxiety, stress and depression [59,61]. Equally, interventions that enhance social opportunities, support systems and amenities at both the village and district levels are critically needed to promote fellowship, cohesion and integration, as well as ensure access to requisite health and social services for the elderly [1,52]. Considering that the GSAP has received little attention in policy or practice, it is imperative for all countries to tackle inequalities in SWB, or face the likely consequences of an older, sicker and more inequitable aging population. 

## Figures and Tables

**Table 1:** Descriptive statistics of selected variables. SD = standard deviation.

Variables	Codes	Men	Women
		Frequency/Mean (%)	Frequency/Mean (%)
**Age (Years)**		65.00 (SD = 10.30, range = 50–90)	64.21(SD = 10.35, range = 50–101)
**Subjective Wellbeing**		7 (SD = 2.81, Range = 0–13.25)	6 (SD = 2.75, Range = 0–12.40)
***Care Responsibilities***			
**Care Responsibilities for Children**		103 (58%) 2.7 (SD = 2.25, Range 0–11)	216 (73%) 3.1 (SD = 2.15, range 0–10)
**Provide Care for Adult**			
No	0	159 (90)	256 (87)
Yes	1	17 (10)	38 (13)
**Receive Family Assistance**			
No	0	96 (55)	106 (36)
Yes	1	80 (45)	188 (64)
**Provide Community Support**			
No	0	111 (63)	192 (65)
Yes	1	65 (37)	102 (35)
**Participate in Group**			
No	0	106 (61)	176 (60)
Yes	1	68 (39)	118 (40)
**Government Assistance**			
No	0	166 (94)	270 (92)
Yes	1	10 (6)	24 (8)
**Receive Remittance from Children**			
Yes	0	32 (18)	106 (36)
No	1	144 (82)	188 (64)
**Number of People Close to (children and adults)**		12 (SD = 19.68, Range 0–100)	8 (SD = 14.45, range 0–98)
***Health care variables***			
**Receive Health Care Last Time Needed**			
No	0	11 (6)	27 (9)
Yes	1	165 (94)	267 (91)
**Length of Waiting time**			
Not long	0	106 (60)	145 (49)
Long	1	70 (40)	149 (51)
**Time to Explain Sickness (HC)**			
Always	0	159 (90)	244 (83)
Not Always	1	17 (10)	50 (17)
**Satisfied with Services (HC)**			
Satisfied	0	158 (93)	241 (88)
Not Satisfied	1	11 (7)	33 (12)
***Socio-economic variables***			
**Asset Level**			
Poor	0	43 (24)	114 (39)
Middle	1	63 (36)	93 (32)
Rich	2	70 (40)	87 (30)
**Financial Status**			
Bad	0	109 (63)	201 (68)
Good	1	64 (37)	93 (32)
**Financial Improvement (last 3yrs)**			
Worse	0	117 (66)	192 (65)
Better/same	1	59 (34)	102 (35)
**Currently Employed/Working**			
No	0	30 (17)	61 (21)
Yes	1	146 (83)	233 (79)
**Educational Level**			
None	0	18 (10)	70 (24)
Primary	1	99 (57)	168 (57)
Secondary	2	47 (27)	38 (13)
Higher	3	9 (5)	18 (6)
***Demographic factors***			
**Marital Status**			
Not Married	0	65 (37)	258 (88)
Married	1	111 (63)	36 (12)
**Residence/Location**			
Urban	0	55 (31)	113 (38)
Rural	1	121 (69)	181 (62)
**Religion**			
Catholic	0	105 (60)	182 (62)
Protestant	1	42 (24)	62 (21)
Islam	2	18 (10)	21 (8)
Other	3	10 (6)	29 (10)
**Total**		**176**	**294**

**Table 2:** Determinants of subjective wellbeing sorted by gender.

Variables	Men only	Women only	Total sample
Age (Years)	0.41[0.27*–*0.54] ***	0.44[0.33*–*0.54] ***	0.42[0.34*–*0.50] ***
Provide Community Support	−0.22[*−*0.39*–−*0.06] ***	*−*0.26[*−*0.39*–−*0.13] ***	*−*0.24[*−*0.34*–−*0.14] ***
Care Responsibilities for Children			
Participate in Group	*−*0.16[*−*0.32*–*0.01] *	*−*0.28[*−*0.41*–−*0.16] ***	*−*0.23[*−*0.33*–−*0.13] ***
Number of People Close to	*−*0.13[*−*0.30*–*0.04]	*−*0.13[*−*0.27*–−*0.002] **	*−*0.15[*−*0.26*–−*0.04] ***
Childcare Responsibilities	*−*0.12[*−*0.28*–*0.05]	*−*0.11[*−*0.24*–*0.03]	*−*0.08[*−*.018*–*0.02]
Provide Care to Adults	0.05[*−*0.11*–*0.23]	*−*0.09[*−*0.23*–*0.04]	*−*0.03[*−*0.14*–*0.07]
Government Assistance	*−*0.04[*−*0.22*–*0.13]	*−*0.29[*−*0.42*–−*0.16] ***	*−*0.19[*−*0.29*–−*0.08] ***
Family Assistance	*−*0.14[*−*0.31*–*0.02] *	0.02[*−*0.11*–*0.16]	0.003[*−*0.10*–*0.11]
Receive Remittance from Children	*−*0.08[*−*0.26*–*0.08]	0.01[*−*0.12*–*0.15]	0.02[*−*0.08*–*0.13]
Receive Healthcare Last Time Needed	0.10[*−*0.07*–*0.27]	*−*0.16[*−*0.29*–−*0.03] **	*−*0.08[*−*0.19*–*0.02]
Length of Waiting Time	*−*0.05[*−*0.23*–*0.12]	0.11[*−*0.03*–*0.24]	0.06[*−*0.04*–*0.17]
Time to Explain Sickness (HC)	0.04[*−*0.13*–*0.22]	0.13[*−*0.01*–*0.26] *	0.12[0.02*–*0.23] **
Satisfied with Services (HC)	*−*0.09[*−*0.26*–*0.08]	0.12[*−*0.01*–*0.25] *	0.07[*−*0.03*–*0.18]
Education	*−*0.31[*−*0.48*–−*0.15] ***	*−*0.23[*−*0.36*–−*0.09] ***	*−*0.29[*−*0.39*–−*0.19] ***
Not Currently Working	0.19[0.03*–*0.35] **	0.28[0.16*–*0.39] ***	0.26[0.17*–*0.35] ***
Asset Level	*−*0.33[*−*0.49*–−*0.17] ***	*−*0.26[*−*0.39*–−*0.13] ***	*−*0.30[*−*0.40*–−*0.20] ***
Good Financial Status	*−*0.47[*−*0.61*–−*0.32] ***	*−*0.44[*−*0.56*–−*0.33] ***	*−*0.45[*−*0.54*–−*0.36] ***
Financial Improvement (last 3 y)	*−*0.40[*−*0.55*–−*0.25] ***	*−*0.46[*−*0.57*–−*0.34] ***	*−*0.42[*−*0.52*–−*0.33] ***
Gender (female)	N/A	N/A	0.21[0.11*–*0.32] ***
Currently Married	*−*0.25[*−*0.42*–−*0.08] ***	*−*0.12[*−*0.25*–*0.02] *	*−*0.26[*−*0.36*–−*0.16] ***
Rural Residence/Location	*−*0.001[*−*0.17*–*0.17]	*−*0.04[*−*0.16*–*0.08]	*−*0.04[*−*0.14*–*0.06]
Religion	0.09[*−*0.08*–*0.26]	0.01[*−*0.13*–*0.14]	0.05[*−*0.06*–*0.16]
RMSEA (Root mean squared error of approximation)			0.097

* *p* < 0.1; ** *p* < 0.05; *** *p* < 0.01. Confidence intervals are presented in brackets. Note: Positive values represent a negative association and negative values represent a positive association. (e.g., Higher values mean worse subjective wellbeing, whereas lower values mean better SWB)
